# Cumulative live birth rates between GnRH-agonist long and GnRH-antagonist protocol in one ART cycle when all embryos transferred: real-word data of 18,853 women from China

**DOI:** 10.1186/s12958-021-00814-0

**Published:** 2021-08-12

**Authors:** Jingwei Yang, Xiaodong Zhang, Xiaoyan Ding, Yuting Wang, Guoning Huang, Hong Ye

**Affiliations:** 1 Chongqing Key Laboratory of Human Embryo Engineering, Chongqing, China; 2 Chongqing Clinical Research Center for Reproductive Medicine, Chongqing, China; 3 Reproductive and Genetic Institute, Chongqing Health Center for Women and Children, No. 64 Jin Tang Street, Yu Zhong District, Chongqing, 400013 China; 4 The Second Affiliated Hospital of Chongqing Medical University, Chongqing, China

**Keywords:** Gonadotropin releasing hormone agonist long protocol, Gonadotropin releasing hormone antagonist protocol, Cumulative live birth rate, ART cycle, Embryo transfer

## Abstract

**Background:**

A consensus has been reached on the preferred primary outcome of all infertility treatment trials, which is the cumulative live birth rate (CLBR). Some recent randomized controlled trials (RCTs) and retrospective studies have compared the effectiveness of GnRH-antagonist and GnRH-agonist protocols but showed inconsistent results. Studies commonly used conservative estimates and optimal estimates to described the CLBR of one incomplete assisted reproductive technology (ART) cycle and there are not many previous studies with data of the complete cycle to compare CLBRs in GnRH-antagonist versus GnRH-agonist protocols.

**Methods:**

A total of 18,853 patients have completed their first IVF cycle including fresh and subsequent frozen-thawed cycles during 2016–2019, 16,827 patients were treated with GnRH-a long and 2026 patients with GnRH-ant protocol. Multivariable logistic analysis was used to evaluate the difference of GnRH-a and GnRH-ant protocol in relation to CLBR. Utilized Propensity Score Matching(PSM) for sampling by up to 1:1 nearest neighbor matching to adjust the numerical difference and balance the confounders between groups.

**Results:**

Before PSM, significant differences were observed in baseline characteristics and the CLBR was 50.91% in the GnRH-a and 33.42% in the GnRH-ant (OR = 2.07; 95%CI: 1.88–2.28; *P* < 0.001). Stratified analysis showed the CLBR of GnRH-ant was lower than GnRH-a in suboptimal responders(46.89 vs 27.42%, OR = 2.34, 95%CI = 1.99–2.74; *P* < 0.001) and no differences of CLBR were observed in other patients between protocols. After adjusting for potential confounders, multivariable logistic analysis found the CLBR of GnRH-ant group was lower than that of GnRH-a group (OR = 2.11, 95%CI:1.69–2.63, *P* < 0.001). After PSM balenced the confounders between groups, the CLBR of GnRH-a group was higher than that of GnRH-ant group in suboptimal responders((38.61 vs 28.22%, OR = 1.60, 95%CI = 1.28–1.99; *P* < 0.001) and the normal fertilization rate and number of available embryo in GnRH-a were higher than these of GnRH-ant groups in suboptimal responders (77.39 vs 75.22%; 2.86 ± 1.26 vs 2.61 ± 1.22; *P* < 0.05). No significant difference was observed in other patients between different protocols.

**Conclusions:**

It is crucial to optimize the utilization of protocols in different ovarian response patients and reconsider the field of application of GnRH-ant protocols in China.

**Supplementary Information:**

The online version contains supplementary material available at 10.1186/s12958-021-00814-0.

## Background

For the past several decades, gonadotropin-releasing hormone agonist (GnRH-a) was the commonly-used modulator to prevent premature LH surge during ovarian stimulation in the process of in-vitro fertilization (IVF)/intracytoplasmic Sperm Injection (ICSI) [1]. Its role in controlled ovarian stimulation (COS) has been remarkable. GnRH-a is competitive in the pituitary gland and block its release of GnRH, thereby inhibiting the secretion of related hormones in the ovary and achieving the effect of pituitary down-regulation [2]. The standard GnRH-a long protocol is the vital one in China due to its association with steady and higher clinical pregnancy rates in fresh embryo transfer (ET) in patients undergoing in vitro fertilization (IVF) [3]. Recently, GnRH antagonist (GnRH-ant) protocol is widely adopted which are more in line with the physiological processes [4]. These advantages include its short treatment duration, a low dose of medication, high compliance of the patients, a low risk of early COS failure [5], quick interaction with the body’s receptors, and reduction in the incidence of severe ovarian hyperstimulation syndrome (OHSS) rate than GnRH-a protocol [6].

Some recent randomized controlled trials (RCTs) and retrospective studies have compared the effectiveness of both protocols but showed inconsistent results [7–10]. So, it is necessary to focus on finding important indicator for making decisions and should be considered as a key point in defining the success of assisted reproductive technology (ART) treatment. This not only reflects the outcome of an embryo transfer, such as pregnancy rate, abortion rate, but also evaluates the potency of all embryos after one oocyte retrieval cycle.

A consensus has been reached on the preferred primary outcome of all infertility treatment trials, which is the live birth rate or cumulative live birth rate (CLBR). It is defined as the live birth per women for over a defined time period (or number of treatment cycles) in 2013 [11]. CLBR requires a long duration period including fresh and subsequent frozen-thawed cycles from the initial oocyte retrieval and there are varying equations to calculate CLBR [12]. Moreover, it might help patients to make decisions for continuing treatment or remaining childless.

Nowadays, the unique medical characteristics of the patients are stored in electronic medical records (EMRs). However, the data from EMRs has less regulatory acceptance as compared to those from RCTs due to weaknesses such as missing information and observational bias, which in turn facilitates the quantity, accessibility, and heterogeneity of offline observational data for a variety of diseases and patients [13].

Hence, this study aimed to compare the CLBR of different number of retrieved oocytes between GnRH-a and GnRH-ant protocol after one complete ART cycle with all embryos used. This approach is advantageous and can be recommended to doctors in choosing an appropriate protocol for patients with more accurate probability of live births.

## Methods

**Fig. 1:**
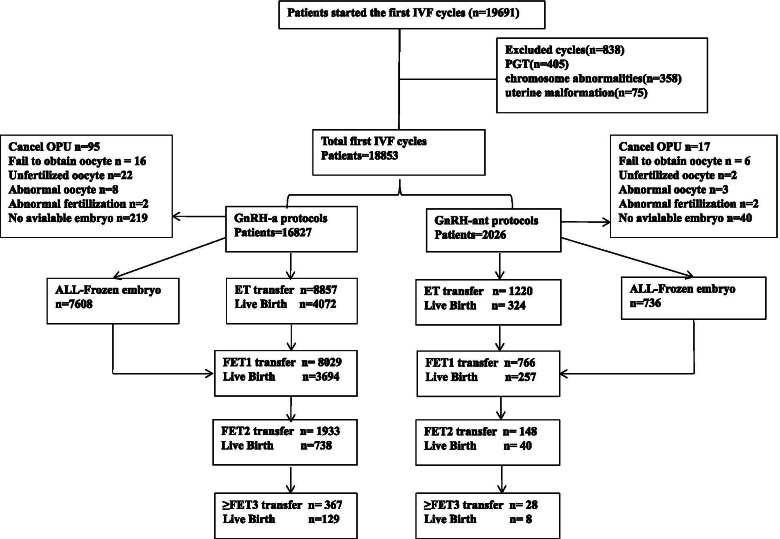
Flow chart

This is a retrospective real-world data study, which links the information of patients from the EMRs, Chongqing Health Center for Women and Children database to evaluate women who commenced their first COS cycle (including IVF and ICSI) including fresh and subsequent frozen-thawed cycles with no embryos left from January 2016 to December 2019. All patients included in this study were undergoing their first COS cycle with GnRH-a protocol or GnRH-ant protocol. Patients with preimplantation genetic testing (PGT) cycles, chromosome abnormalities and uterine malformation were excluded. Finally, a total of 18,853 patients were analyzed, and 16,827 patients underwent treatment with GnRH-a long protocol and 2026 patients underwent treatment with GnRH-ant protocol (Fig. 1).

The study involving human participants was reviewed and approved by the Ethics Committee Review Board of Chongqing Health Center for Women and Children (approval number: 2020-RGI-13) for retrospective analysis and clinical data reporting. Informed consent was waived by the committee because of the retrospective nature of the study.

### GnRH-agonist long protocol

GnRH-a (Triptorelin 0.1 mg/d or 0.05 mg/d, sc. Decapeptyl Ferring, Germany) was used for pituitary downregulation from the previous luteal phase. After administration of GnRH agonist for 14–21 days, if the levels of estrogen < 50 pg/mL, luteinizing hormone < 5 mIU/mL and P < 1 ng/mL, then a dose of recombinant follicle stimulating hormone (rFSH) ranging from 75 to 300 IU was administrated subcutaneously per day based on the woman’s age, antimüllerian hormone (AMH) level and antral follicle counts (AFC).

### GnRH-antagonist protocol

COS was initiated on day 2 or 3 of the cycle with a dose of rFSH ranging from 75 to 300 IU. GnRH antagonist of 0.25 mg (Orgalutran, Organon, The Netherlands or Cetrorelix, Merck serono, Switzerland) was given to patients daily if at least one of the following criteria was fulfilled: (i) with at least one follicle of > 14 mm; (ii) serum estrogen level of > 600 pg/mL; and (iii) serum LH level of > 10 IU/L [14].

### Trigger day

If there were at least three follicles were measured > 18 mm in diameter, human chorionic gonadotropin (hCG, Merck Serono, Italy) was administered and part of GnRH-ant cycles used Triptorelin Acetate Injection (GnRH-a, Ferring GmbH, Germany) as trigger. After that, transvaginal oocyte retrieval was performed 36 h, and then embryo transfer (ET) was performed on day 3 after oocyte retrieval. Luteal-phase support was started immediately after oocyte retrieval with vaginal combined oral progesterone. Most of the patients received double embryo transfer (DET) unless the patient had only one available embryo for single embryo transfer according to the national guidelines [15]. Surplus available embryos or all-frozen embryos (due to OHSS, thin endometrium, abnormal blood biochemical index and personal causes of patients) were frozen for later transfer in subsequent frozen embryo transfer (FET) cycles. The vast majority of these embryos were frozen on day 3. Embryos that are not suitable for cryopreservation on day 3 were cultured till days 5 or 6 and vitrified if they reach the blastocyst stage. Luteal-phase support with vaginal combined oral progesterone was started three days before FET.

### Vitrification and storage

The cryotop vitrification method [the Kitazato vitrification kit (Kita, Toyota, Japan)] were used in all procedures and the vitrification procedure was carried out according to the manufacturer's instructions as reported by Kuwayama et al. [16]. The solution used as a basic media was 4-[2-hydroxyethyl]-1-piperazine ethane sulfonic acid (HEPES)-buffered human tubal fluid (HTF) + 20% Serum protein substitute (SPS;Sigma). Vitrification was performed at room temperature (23–25℃) and embryos were first suspended in equilibration solution (VS1)[VS1 = 7.5% (v/v) ethylene glycol (EG;Sigma) + 7.5%(v/v) dimethylsulfoxide (DMSO;Sigma)] for a 8-12 min equilibration time for vitrification. Then, embryos were exposed to the vitrification solution (VS2)[VS2 = 15.0% (v/v) EG + 15.0% (v/v) DMSO + 0.5 M sucrose(Sigma)] for 45-60 s. Finally, embryos were loaded on the tip of Cryotop and excess vitrification solution was removed by aspiration using a pipette before the immersion of the carrier in liquid nitrogen (LN_2_) immediately.

### Warming procedure

A Dewar of LN_2_ containing the carriers was placed close to the microscope. Forceps were used to grasp the straw in the LN_2_ and placed it in a dish containing 3 mL of 1.0 mol/L sucrose at 37 °C for 1 min. All the embryos were then transferred sequentially to 0.5 and 0.25 mol/L sucrose solutions at room temperature for 3 min each. The embryos were then washed several times in Quinn’s 1024 (Cooper Surgical, CT, USA) solution, and placed in G1 medium (Vitrolife, Kungsbacka, Sweden) for further culturing. Post-warming survival of cryopreserved embryos was defined as survival of more than one-half of the original cells that are intact.

If pregnancy is achieved, then luteal phase support was continued until 12 weeks’ gestation in both the groups.

### Outcome measures

The indexes of embryo quality were the D2-4c (Day 2–4 cell) rate and D3-8c (Day 3–8 cell) rate defined as the proportion of embryos with 4/8 cells on Day 2/3 of the total number of two pronuclei (2PN) cleavege embryos. The primary outcome was the cumulative live birth per Ovum Pick-Up (OPU), which was defined as the first live born baby at ≥ 28 weeks’ gestation that results from a completed ART cycle, including all fresh and FETs that result from the associated ovarian stimulation. If a live birth occurs, then the patients can obtain an outcome regardless of subsequent cycles. According to this definition, multiple deliveries from the same pregnancy or multiple live births were considered as one live birth. CLBRs were calculated as the proportion of cycles that achieved the first live birth.

### Statistics

Data are presented as means (SD) or number (%) as appropriate. Wilcoxon rank-sum test was used for analyzing continuous variables. The cumulative pregnancy rates and CLBRs in the GnRH-a and GnRH-ant groups were compared by Chi-square test. The number of oocytes retrieved were categorized into five groups, namely 1–3 (poor), 4–9 (suboptimal), 10–15 (normal) and > 16 (high) [17]. The Cochran-Mantel–Haenszel (CMH) test by different levels of retrieved oocytes was used to compare the CLBR, normal fertilization rate, D2-4c rate and D3-8c rate between protocol groups. A multivariable logistic regression analysis were used to evaluate the relative prognostic significance of protocol groups, female age, body mass index (BMI), AMH, FSH, the number of retrieved oocytes, the number of available embryos, type of infertility in relation to CLBR. Interactions between independent covariates were adjusted. Utilized Propensity Score Matching (PSM) for sampling by up to 1:1 nearest neighbor matching with caliper (0.05) to balence the baseline and improve the comparability between groups. *P*-values of < 0.05 were considered to indicate statistical significance. All analyses were conducted using Stata (version 15.1, College Station, TX: StataCorp LLC).

## Results

Following planned exclusions, 18,853 women were included in this analysis (Fig. 1). Epidemiological, clinical, and biological characteristics of the population before and after PSM are summarized in Table 1. Before PSM, significant differences were observed between the two protocols in age, infertility duration, BMI, AMH, FSH, Gn dose, Gn day, retrieved oocytes, available embryos, type of infertility. After 1:1 nearest neighbor PSM matching, the analysis focused on 3439 patients (1704 patients in GnRH-a group and 1735 patients in GnRH-ant group) and the mean age of the study population was 34.06 ± 4.94 years. No differences in age, infertility duration, BMI, AMH, FSH, Gn dose, available embryos, cause of infertility between groups. Significant differences between the two comparative groups were observed in Gn days (10.14 ± 1.61 in GnRH-a vs 9.89 ± 1.58 in GnRH-ant groups; *P* < 0.001), number of oocytes retrieved(10.43 ± 6.28 in GnRH-a vs 9.68 ± 7.11 in GnRH-ant groups; *P* < 0.001) and available embryo(3.92 ± 2.69 in GnRH-a vs 3.68 ± 2.76 in GnRH-ant groups; *P* < 0.001). Patient assignment before and after PSM is reported in Supplemental Figure 1.

**Table 1:** Baseline characteristics of women at first IVF cycle treated with either GnRH-agonist or GnRH-antagonist protocol and after PSM

	Unmatch / Matched	GnRH-a (Unmatch *n* = 16,827; Matched *n* = 1704)	GnRH-ant (Unmatch *n* = 2026; Matched *n* = 1735)	*P*
Age at oocytes retrieval (years)	U	30.88 ± 4.05(30.81–31.07)	34.33 ± 5.71(33.95–34.61)	< 0.001
	M	34.10 ± 4.11(34.07–34.57)	34.02 ± 5.64(33.56–34.23)	0.392
Duration of infertility (years)	U	4.9 ± 3.53(4.84–4.98)	5.73 ± 4.56(5.38–5.88)	< 0.001
	M	5.83 ± 4.41(5.51–6.04)	5.70 ± 4.49(5.29–5.81)	0.233
BMI (kg/m^2^)	U	21.94 ± 2.81(21.93–22.06)	22.37 ± 2.76(22.25–22.56)	< 0.001
	M	22.38 ± 2.81(22.27–22.61)	22.28 ± 2.75(22.17–22.49)	0.601
AMH (ng/mL)	U	3.72 ± 2.81(3.70–3.81)	3.34 ± 2.26(3.31–3.92)	< 0.001
	M	3.55 ± 3.23(3.36–3.80)	3.45 ± 3.29(3.38–4.04)	0.481
FSH (mIU/mL)	U	5.5 ± 3.36(5.40–5.54)	7.08 ± 4.88(5.90–8.20)	< 0.001
	M	5.72 ± 1.69(5.53–5.73)	7.08 ± 4.09(5.84–8.32)	0.013
Gn days	U	10.78 ± 1.45(10.77–10.83)	9.72 ± 1.66(9.71–9.90)	< 0.001
	M	10.14 ± 1.61(10.05–10.22)	9.89 ± 1.58(9.85–10.03)	< 0.001
Gn dose (mg)	U	2433.67 ± 824.98(2398.80–2489.95)	2255.09 ± 767.56(2246.95–2277.30)	< 0.001
	M	2490.99 ± 785.37(2481.58–2574.43)	2468.88 ± 844.48(2402.04–2499.23)	0.614
Retrieval oocytes	U	12.94 ± 6.33(12.90–13.15)	9.19 ± 7.23(9.03–9.82)	< 0.001
	M	10.43 ± 6.28(10.17–10.92)	9.68 ± 7.11(9.37–10.09)	< 0.001
Available embryo	U	4.62 ± 2.82(4.52–4.63)	3.66 ± 2.78(3.44–3.75)	< 0.001
	M	3.92 ± 2.69(3.75–4.09)	3.68 ± 2.76(3.52–3.85)	< 0.001
Cause of infertility				
Primary infertility	U	7497/16827(44.55%)	735/2026(36.28%)	< 0.001
	M	601/1704(35.27%)	641/1735(36.95%)	0.320
Secondary infertility	U	9333/16827(55.46%)	1291/2026(63.72%)	
	M	1103/1704(64.73%)	1094/1735(63.05%)	
IVF/ICSI				
IVF	U	13,462/16708(80.57%)	1648/1974(83.48%)	0.002
	M	1398/1704(82.04%)	1433/1735(82.59%)	0.688
ICSI	U	3246/16708(19.43%)	326/1974(16.52%)	
	M	306/1704(17.96%)	302/1735(17.41%)	

The CLBR after one complete ART cycle was 8567/16827 (50.91%) in the GnRH-a group and 677/2026 (33.42%) in the GnRH-ant group (OR = 2.07; 95% CI: 1.88–2.28; *P* < 0.001). Stratified analysis of CLBR was performed after grouping number of oocytes retrieved. There were higher CLBR in GnRH-a than GnRH-ant in poor responders (25.72 vs 18.13%, OR = 1.56, 95%CI = 1.13–2.16, *P* = 0.007) and suboptimal responders (46.89 vs 27.42%, OR = 2.34, 95%CI = 1.99–2.74; *P* < 0.001). After 1:1 nearest neighbor PSM matching, the CLBR was 689/1704 (40.43%) in the GnRH-a group and 621/1735 (35.79%) in the GnRH-ant group (OR = 1.22; 95% CI: 1.06–1.40; *P* = 0.006). In CMH stratified analysis, there were significant differences in CLBR which of GnRH-a group was higher than that of GnRH-ant group in suboptimal responders (38.61 vs 28.22%, OR = 1.60, 95%CI = 1.28–1.99; *P* < 0.001). No differences in CLBR between protocols in other subgroup(Table 2).

**Table 2:** Stratified analysis of CLBR after grouping age and number of retrieval oocytes

Age	Before PSM		After PSM	
Ovarian response	GnRH-a	GnRH-ant	GnRH-a	GnRH-ant
No embryo to transfer	362	70	87	36
Poor	151/587(25.72%)*	68/375(18.13%)*	29/150(19.33%)	60 /314(19.11%)
Suboptimal	2194/4679(46.89%)*	241/879(27.42%)*	266/689(38.61%)*	217/769(28.22%)*
Normal	3370/6105(55.20%)	185/356(51.97%)	232/467(49.68%)	176/317(55.52%)
High	2852/5094(55.99%)	183/346(52.89%)	162/311(52.09%)	168/299(56.19%)

^*^ Before PSM, poor responders: GnRH-a:GnRH-ant, OR = 1.56, 95%CI = 1.13–2.16, *P* = 0.007

^*^ Before PSM, suboptimal responders: GnRH-a:GnRH-ant, OR = 2.34, 95%CI = 1.99–2.74, *P* < 0.001

^*^After PSM, suboptimal responders: GnRH-a:GnRH-ant, OR = 1.60, 95%CI = 1.28–1.99, *P* < 0.001

Considering the results of stratified analysis, multivariable logistic analysis based on the four subgroups of ovarian response (poor; suboptimal; normal; high) was performed. After adjusting for potential confounders (such as age, infertility duration, BMI, AMH, FSH, available embryos, cause of infertility, IVF/ICSI) in different ovarian responders, the protocol acts as an independent influential factor of CLBR for suboptimal ovarian responders. Significant difference was found the CLBR of GnRH-ant group was lower than that of GnRH-a group (OR = 2.11, 95%CI:1.69–2.63; *P* < 0.001). In addition, age and available embryo were the important factors for each subgroups (Table 3). After PSM matching, adjusting for potential confounders (age, FSH, available embryo, retrieval oocyte and Gn day) in different ovarian responders, the protocol acts as an independent influential factor of CLBR for suboptimal ovarian responders (OR = 1.84, 95%CI:1.37–2.47; *P* < 0.001) (Supplemental Table 1).

**Table 3:** Multivariable logistic regression of CLBR in different oocyte retrieval groups after adjusting for potential confounders (Before PSM)

Variables	Poor	Suboptimal	Normal	High
Group (1 = GnRH-agonist; 2 = GnRH-antagonist)	1.15(0.70–1.88)	2.11(1.69–2.63)*	1.04 (0.78–1.38)	1.01(0.98–1.03)
Age	0.89(0.84–0.94)*	0.93(0.91–0.94)*	0.93(0.92–0.95)*	0.96(0.94–0.98)*
AMH	0.91(0.78–1.07)	1.00(0.97–1.04)	1.02 (0.99–1.04)	1.06(1.04–1.09)*
BMI	0.97(0.90–1.04)	0.98(0.95–1.01)	0.96(0.94–0.99)*	0.99(0.97–1.02)
Available Embryo	1.47(1.02–2.12)*	1.32(1.25–1.39)*	1.19(1.14–1.23)*	1.10(1.07–1.13)*
IVF/ICSI	0.93(0.46–1.85)	1.08(0.88–1.33)	1.20(1.01–1.44)*	1.39(1.14–1.66)*

Results showing the parameters with significant differences after adjusting for potential confounders (such as age, infertility duration, BMI, AMH, FSH, available embryo, cause of infertility and IVF/ICSI)

^*^P < 0.05

After 1:1 nearest neighbor PSM matching, by stratified retrieval oocyte, the analysis of embryo quality showed that the normal fertilization rate and number of available embryo in GnRH-a were higher than these of GnRH-ant groups in suboptimal responders (77.39 vs 75.22%; 2.86 ± 1.26 vs 2.61 ± 1.22; *P* < 0.05). No significant difference was observed in normal fertilization rate and D2-4c rate, D3-8c rate between different protocols (Table 4).

**Table 4:** Embryo quality of patients treated with GnRH-a or GnRH-ant protocol(After PSM)

Ovarian Response	Available embryo		Normal fertilization rate		D2-4c rate		D3-8c rate	
	GnRH-a	GnRH-ant	GnRH-a	GnRH-ant	GnRH-a	GnRH-ant	GnRH-a	GnRH-ant
Poor	1.55 ± 0.56	1.56 ± 0.58	221/294(75.17)	472/639(73.86)	127/219(57.99)	274/457(59.96)	89/219(40.64)	162/457(35.44)
Suboptimal	2.86 ± 1.26^a^	2.61 ± 1.22^b^	3115/4025(77.39)*	3076/4089(75.22)*	1847/3034(60.88)	1814/2984(60.79)	1200/3034(39.55)	1109/2984(37.16)
Normal	4.41 ± 2.01	4.50 ± 2.20	3894/5036(77.32)	2635/3430(76.82)	2250/3777(59.57)	1559/2554(61.04)	1421/3777(37.62)	987/2554(38.64)
High	7.08 ± 3.36	7.11 ± 3.39	4318/5658(76.32)	4315/5630(76.64)	2595/4228(61.37)	2498/4211(59.32)	1430/4228(33.82)	1377/4201(32.78)

^a^ vs ^b^: 2.86 ± 1.26 vs 2.61 ± 1.22; *P* < 0.001

^*^: GnRH-a:GnRH-ant, OR(95%CI) = 1.127(1.018–1.249); *P* = 0.023

## Discussion

GnRH-ant protocol is widely used because of its short treatment duration, and lower gonadotropin requirement [6]. This protocol offers an available alternative to the long agonists, providing a shorter duration of treatment with fewer injections and with no adverse effects on ART outcome [18]. Our study showed that GnRH-ant protocol involves less Gn days [(9.72 ± 1.66)d vs (10.78 ± 1.45)d] as well as Gn dose [(2255.09 ± 767.56)IU vs (2433.67 ± 824.98)IU] than GnRH-a protocol. In addition, OHSS is a preventable condition and implementation of evidence-based prevention strategies enables clinicians to reduce its occurrence, and there are many evidences showing significantly lower rate of OHSS in GnRH-ant protocol [19–21].

Considering these outcomes, it is still controversial on the efficacy of GnRH-a and GnRH-ant protocols. The main finding of this study was that the CLBR was comparable in both GnRH-a and GnRH-ant groups, with 50.91 and 33.42% achieving the first live birth. After adjusting for potential confounders, the CLBR of GnRH-ant group was lower than that of GnRH-a group in suboptimal ovarian responders. But for other patients, no difference in CLBR was found between the two protocols. The CLBR presented in this study was based on previous studies, which showed lower pregnancy rate, ongoing pregnancy rate and LBR of fresh transfer cycles in the GnRH-ant protocol than in the GnRH-a protocol [3]. While other studies showed no significant difference in clinical pregnancy rates, ongoing pregnancy rate and LBR between GnRH-a protocol and GnRH-ant protocol [6, 22, 23].

A RCT by M.Toftager et al. [10] showed no difference in baseline characteristics, and revealed CLBR to be 34.1% in the GnRH-ant group versus 31.2% in the GnRH-a group (OR:1.14; 95% CI: 0.88–1.48, *P* = 0.32). Though there was a difference with our study results, the presence of population differences is a negligible issue. Indeed, pivotal RCTs often lacked external validity as eligibility criteria excluded some of the patient subgroups who were commonly treated in real-world clinical practice [24]. In China, by considering the financial burden of patients, and in order to improve the success rate of each ET, the GnRH-a protocol is still regarded as the main protocol but the comfort of treatment was not taken into account. The GnRH-ant protocol was mostly recommended for patients who are more prone to OHSS. The population differences demonstrated an effect on objective comparison of two different treatment conditions. The impact of age and oocytes retrieval on CLBR has been confirmed in several prospective studies published in recently [25–27]. Other studies have found that the retrieved oocytes showed positive association with the number of euploid embryos that are available for transfer. These findings provide an explanation with regard to higher availability of euploid embryos for transfer, resulting in increased CLBR from higher oocyte yields [28, 29].

Selecting an ovarian stimulation protocol for patients with poor ovarian response is difficult because of the retrieval of fewer oocytes. Huang et al. [8] have observed that the GnRH-a protocol showed correlation with higher LBR and implantation rate than the GnRH-ant protocol for ET cycles in patients with diminished ovarian reserve (DOR), suggesting that only a few oocytes can be retrieved from these patients in a single stimulation cycle. The LBR and implantation rate showed association with endometrial and embryo quality. In our results, due to the differences of normal fertilization rate and available embryo but no difference of embryo quality, unsynchronization of follicle development [30] and decreased endometrial receptivity of GnRH-ant protocol might be related to the clinical outcomes [31, 32].

There is asynchrony in antral follicle development during early follicular phase [33] which is further expanded in progress of COS. In the ovary, AMH is produced by the granulosa cells of early developing follicles and seems to be able to inhibit the initiation of primordial and FSH-induced follicle growth. Wang B et al.speculated that in addition to the flare up effect of GnRH-a, GnRH-a could reduce the AMH expression in small antral folliclea which increase the responsiveness to FSH and promote growth, however, the effect on larger follicles is not obvious [30, 34]. Other researches provided the new evidence that GnRH-a and GnRH-ant showed different effect on ovarian reserve and suggest that this discrepancy might be caused by different regulation on intraovairan autocrine/paracrine factors AMH and SCF through GnRH-I system [35]. Hernandez et al. have shown that GnRH-ant might disrupt an auto/paracrine loop that is essential for mitotic program of the endometrial cells and it is manifested by a decrease in the pregnancy rates and increase in the abortion rates [36]. Rackow et al. have found HOXA10 (an essential regulator of endometrial receptivity) expression was significantly decreased in endometrial stromal cells in GnRH-ant treated cycles when compared with GnRH-a treated cycles or natural cycle controls [32]. Ruan et al. have found that GnRH-a, but not GnRH antagonist, have partially restored the endometrial physiological secretion and improved uterine receptivity in mice [37]. A comparative proteomic analysis demonstrated that endometrial receptivity is more strongly impaired by GnRH-ant than GnRH-a protocols [38]. The results of the above studies indicated that the endometrial receptivity of GnRH-a protocol might be better than GnRH-ant protocol.

For normal and high ovarian responder patients, GnRH-ant protocol group had lower levels of Gn dosage, days of Gn administered, number of retrieved oocytes, number of matured oocytes, normal fertilization rate [39] and substantial reduction in OHSS without reducing the likelihood of achieving live birth or ongoing pregnancy [22]. In our study, all subgroup analyses for covariate's effect on the CLBR were based on post hoc analyses with multiple strata. Female age, IVF/ICSI and available embryo were the influential factors of CLBR in normal and high ovarian responder patients and the protocols showed no effect on the CLBR. From one OPU involves multiple transfer cycles and a very few patient received single embryo transfer, we found it had no effect on the CLBR. Wen Ding et al. [40] have confirmed that female obesity has adversely affected the CLBR after utilizing the available embryos from first oocyte retrieval. But we found BMI as a unique influential factor in only normal ovarian responder patients, and so it should be interpreted with caution and findings should be further investigated.

However, this study has limitation due to its retrospective nature. Due to analysis of real-world data, the characteristics of patients were not balanced. Although the confounders were adjusted using mutivariable logistic regression and PSM for sampling to balence the baseline between groups, some potential confounders might be neglected. A well-designed, multicenter, prospective RCT is still warranted to further support our results. Different molecular mechanisms of endometrial receptivity between GnRH-a and GnRH-ant protocols should be evaluated for further study.

After one complete cycle, despite significantly higher CLBR in the GnRH-a protocol for suboptimal ovarian response patients, no significant difference of CLBR was observed between these two protocols in other patients. However, it is crucial to optimize the utilization of protocols in different ovarian response patients, personalized protocols for patients and reconsider the field of application of GnRH-ant protocol in China.

## Conclusion

To provide evidence for protocol choosing, under a large sample study, our results suggest that despite significantly higher CLBR in the GnRH-a protocol for suboptimal ovarian response patients, no significant difference of CLBR was observed between these two protocols in other patients. It is crucial to optimize the utilization of protocols in different ovarian response patients, personalized protocols for patients and reconsider the field of application of GnRH-ant protocol in China. Given the unbalanced characteristics of patients, though the confounders were adjusted, the results should be further validated by well-designed RCTs.

## Supplementary Information

**Additional file 1: Supplemental Table 1.** Multivariable logistic regression of CLBR in different oocyte retrieval groups after adjusting for potential confounders (After PSM). **Supplemental Figure 1.** Study flow chart before and after PSM

## Data Availability

The datasets used and/or analyzed during the current study are available from the corresponding author on reasonable request.

## Abbreviations

ART: Assisted reproductive technology
AMH: Anti-Müllerian hormone
AFC: Antral follicle count
BMI: Body mass index
COS: Controlled ovarian stimulation
CLBR: Cumulative live birth rate
ET: Embryo transfer
EMRs: Electronic medical records
FET: Frozen-embryo transfer
GnRH-a: Gonadotropin-releasing hormone agonist
GnRH-ant: Gonadotropin-releasing hormone antagonist
IVF: In vitro fertilization
ICSI: Intracytoplasmic sperm injection
OHSS: Ovarian hyperstimulation syndrome
OPU: Ovum-pick up
OCP: Oral contraceptive pill
POR: Poor ovarian response
PGT: Preimplantation genetic testing
PSM: Propensity Score Matching
RCTs: Randomized controlled trials
rFSH: Recombinant follicle-stimulating hormone
