# Supplementary material for: Cumulative live birth rates between GnRH-agonist long and GnRH-antagonist protocol in one ART cycle when all embryos transferred: real-word data of 18,853 women from China
Source: Reprod Biol Endocrinol. 2021 Aug 12;19:124. doi: 10.1186/s12958-021-00814-0 (PMC8359059; doi:10.1186/s12958-021-00814-0)

Supplemental Table1

Multivariable logistic regression of CLBR in different oocyte retrieval groups after adjusting for potential confounders(After PSM)

| Variables | Poor | Suboptimal | Normal | High |
| --- | --- | --- | --- | --- |
| Group (1=GnRH-agonist; 2=GnRH-antagonist) | 0.98(0.51-1.92) | 1.84(1.37-2.47)* | 0.95 (0.65-1.38) | 1.13(0.73-1.73) |
| Age | 0.83(0.76-0.90)* | 0.91(0.88-0.94)* | 0.91(0.87-0.94)* | 0.94(0.90-0.99)* |
| FSH | 0.99(0.91-1.10) | 1.02(0.96-1.08) | 1.02(0.94-1.11) | 1.09(0.91-1.31) |
| Gn day | 0.92(0.56-1.55) | 1.03(0.93-1.14) | 0.95(0.83-1.10) | 0.97(0.83-1.13) |
| Retrieval oocyte | 1.38(0.79-2.42) | 1.05(0.95-1.15) | 0.94(0.84-1.06) | 0.99(0.95-1.04) |
| Available Embryo | 1.04(0.35-3.04) | 1.37(1.23-1.54)* | 1.17(1.07-1.28)* | 1.07(1.01-1.13)* |

Results showing the parameters with significant differences after adjusting for potential confounders (such as age, FSH, available embryo, retrieval oocyte and Gn day).

**P*<0.05

Supplemental Figure 1 Study flow chart before and after PSM


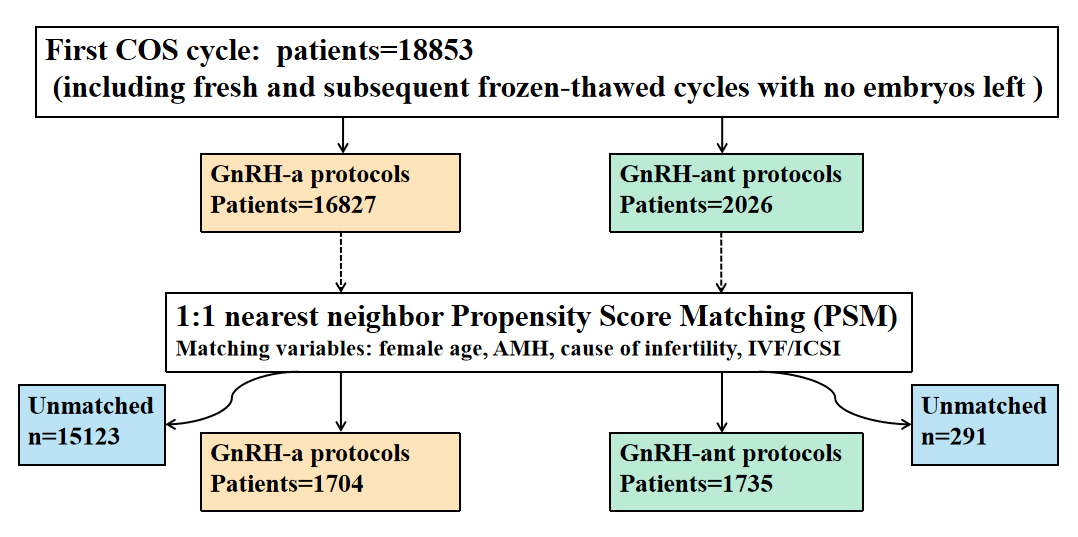

Supplement: Supplementary file 1 — Additional file 1: Supplemental Table 1. Multivariable logistic regression of CLBR in different oocyte retrieval groups after adjusting for potential confounders (After PSM). Supplemental Figure 1. Study flow chart before and after PSM [file 12958_2021_814_MOESM1_ESM.doc]
